# MOD-4023, a long-acting carboxy-terminal peptide-modified human growth hormone: results of a Phase 2 study in growth hormone-deficient adults

**DOI:** 10.1530/EJE-16-0748

**Published:** 2016-12-08

**Authors:** Christian J Strasburger, Peter Vanuga, Juraj Payer, Marija Pfeifer, Vera Popovic, László Bajnok, Miklós Góth, Veˇra Olšovská, L‘udmila Trejbalová, Janos Vadasz, Eyal Fima, Ronit Koren, Leanne Amitzi, Martin Bidlingmaier, Oren Hershkovitz, Gili Hart, Beverly M K Biller

**Affiliations:** 1 Department of Medicine for EndocrinologyDiabetes and Nutritional Medicine, Charité Universitätsmedizin, Berlin, Germany; 2 Department of EndocrinologyNational Institute of Endocrinology and Diabetology, Lubochna, Slovakia; 3 Department of Internal Medicine VUniversity Hospital Ruzinov, Bratislava, Slovakia; 4 Department of EndocrinologyUniversity Medical Centre Ljubljana, Ljubljana, Slovenia; 5 Neuroendocrine UnitClinical Centre of Serbia, Belgrade, Serbia; 6 1st Department of MedicineUniversity of Pécs, Pécs, Hungary; 7 2nd Department of Internal MedicineMilitary Hospital – State Health Center, Budapest, Hungary; 8 II Internal Clinic in University Hospital St AnnaBrno, Czech Republic; 9 I Department of Internal MedicineUniversity Hospital Bratislava, Bratislava, Slovakia; 10 1st Department of Internal MedicineHetényi Géza Hospital and Out-Patient Clinic, Szolnok, Hungary; 11 OPKO BiologicsKiryat Gat, Israel; 12 Medizinische Klinik – InnenstadtLudwig Maximilian University, Munich, Germany; 13 Neuroendocrine UnitMassachusetts General Hospital, Boston, Massachusetts, USA

## Abstract

**Objective:**

Growth hormone (GH) replacement therapy currently requires daily injections, which may cause distress and low compliance. C-terminal peptide (CTP)-modified growth hormone (MOD-4023) is being developed as a once-weekly dosing regimen in patients with GH deficiency (GHD). This study’s objective is to evaluate the safety, pharmacokinetics (PK), pharmacodynamics (PD) and efficacy of MOD-4023 administered once-weekly in GHD adults.

**Design:**

54 adults with GHD currently treated with daily GH were normalized and randomized into 4 weekly dosing cohorts of MOD-4023 at 18.5%, 37%, 55.5% or 123.4% of individual cumulative weekly molar hGH dose. The study included 2 stages: Stage A assessed the effectiveness and PK/PD profiles of the 4 dosing regimens of MOD-4023. Stage B was an extension period of once-weekly MOD-4023 administration (61.7% molar hGH content) to collect further safety data and confirm the results from Stage A.

**Results:**

Dose-dependent response was observed for both PK and PD data of weekly MOD-4023 treatment. Insulin-like growth factor I (IGF-I) SDS levels were maintained within normal range. The 18.5% cohort was discontinued due to low efficacy. MOD-4023 was well tolerated and exhibited favorable safety profile in all dose cohorts. The reported adverse events were consistent with known GH-related side effects.

**Conclusions:**

Once-weekly MOD-4023 administration in GHD adults was found to be clinically effective while maintaining a favorable safety profile and may obviate the need for daily injections. Weekly GH injections may improve compliance and overall outcome. The promising results achieved in this Phase 2 study led to a pivotal Phase 3 trial, which is currently ongoing.

## Introduction

The primary goal of growth hormone (GH) replacement in adults is to correct the abnormalities associated with GH deficiency (GHD). Current guidelines for the use of r-hGH in adults recommend individualized dosing based on serum IGF-I levels and the absence of adverse events (AEs) (1, 2, 3). The demonstrated benefits of GH therapy include improvement in body composition, surrogate markers of cardiovascular risk factors, exercise capacity, skeletal integrity and quality of life (1, 2, 3, 4,  5, 6).

GH treatment currently requires administration using daily subcutaneous injections (1, 3). However, adherence to this regimen has been shown to be suboptimal (7, 8). This is important as GH replacement is required not only for growth during childhood but also for metabolic health during adulthood (9). Long-term continuous GH exposure in adult patients with GHD produced comparable responses in terms of bone metabolism, body composition, insulin sensitivity and lipid metabolism as with daily GH injections (10, 11). A GH treatment regimen that requires less frequent injections may improve adherence and, potentially, overall outcomes (12). A variety of strategies have been employed to prolong the duration of GH action, and several are currently undergoing clinical trials (13, 14).

Carboxy-terminal peptide (CTP) technology (OPKO Biologics) has enabled the production of a long-acting hGH (MOD-4023), which may obviate the need for the numerous injections now required for the treatment of GHD in children and adults. CTP technology is based on a natural peptide, derived from the C-terminus of human chorionic gonadotropin (hCG (15)), which prolonged the half-life and increased the bioavailability of proteins such as FSH (16) and erythropoietin (17). hGH-CTP (MOD-4023) was shown in animal models to have a significantly longer half-life than r-hGH (18) and a favorable safety profile in a Phase 1 clinical study conducted in healthy volunteers (19). Based on these findings, MOD-4023 has the potential to be injected once per week, as opposed to the current need for daily hGH injections.

The present investigation (ClinicalTrials.gov identifier Nbib1225666) was designed as a dose-finding study to assess the safety, tolerability and PK/PD parameters of an individualized dose of MOD-4023 in adult GHD patients switched from ongoing daily GH injections to less frequent dosing.

## Subjects and methods

### Subjects

Eligible patients for this study were males between 23 and 60 and females between 23 and 50 years of age, defined as GHD according to the 2007 Consensus Guidelines (2), who had taken GH replacement therapy for more than 6 months. Because the normal range for IGF-I varies with age, the entry criterion for patients’ enrollment was set at a standard deviation score (SDS) target range of ±1.5 IGF-I for gender and chronological age, to minimize data variability. The eligibility criterion for body mass index (BMI) was between 19 and 35 kg/m^2^. All patients were confirmed as negative for anti-GH antibodies at screening. Patients were excluded from the study if they had evidence of growth of pituitary adenoma or other intracranial tumors, history of malignancy, intracranial hypertension, significant heart failure, impaired liver or kidney function, active acromegaly, glucocorticoid therapy or syndromes such as Prader–Willi or Cushing’s. All study subjects provided written informed consent prior to study initiation.

### Study design

**Figure 1:**
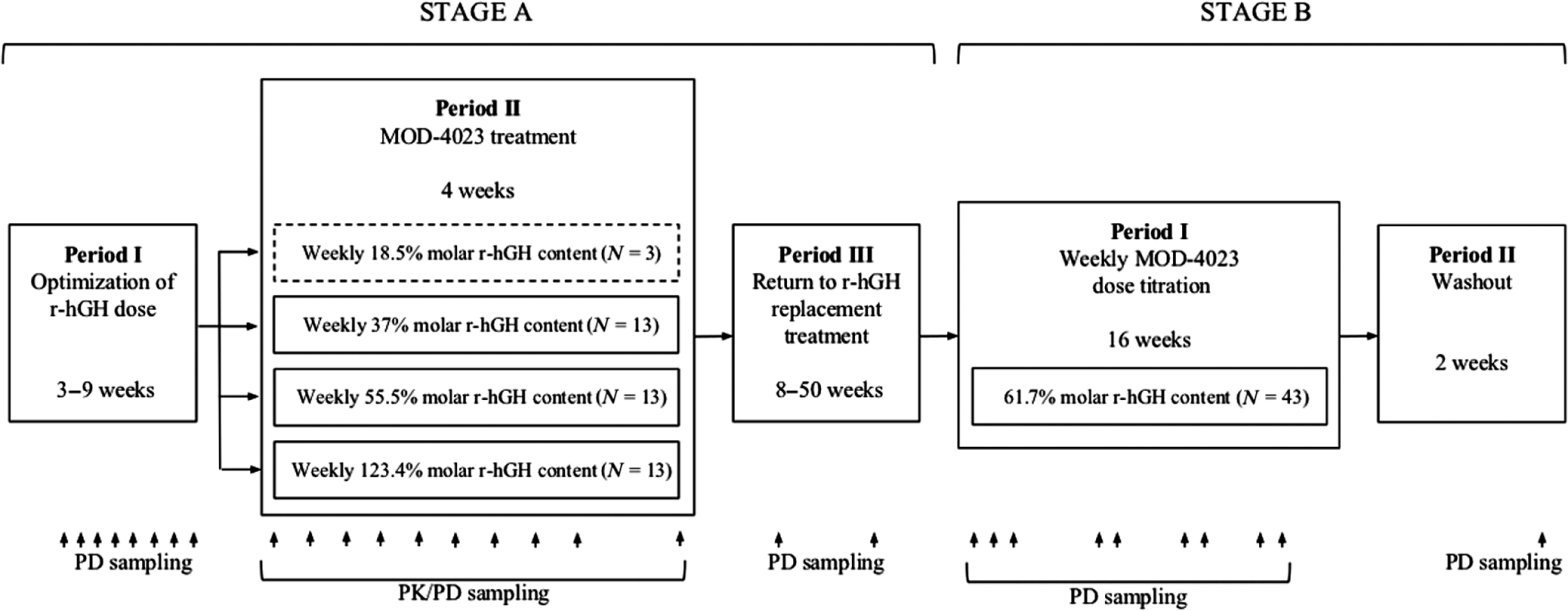
Overview of study design. The two stages of the study are shown, divided into five specific periods. MOD-4023 doses are presented as % of the patients’ cumulative weekly r-hGH dose. For MOD-4023 dosing during Stage A, Period II, the reference period was Stage A, Period I. For dosing in Stage B, the reference period was Stage A, Period III. The PK and PD sampling time points are shown along the bottom of the figure. The discontinued cohort is framed in a dashed line.

This was a multicenter, multi-dose, open-label dose-finding Phase 2 study to evaluate the safety, tolerability and PK/PD profile of MOD-4023 administered subcutaneously to GHD adults on daily hGH therapy. The protocol was reviewed and approved by the Ethics committees of all study sites, and written informed consent was obtained from all patients participating in the study. The study began with a screening period to assess patient’s eligibility for the study. After the screening period (up to 21 days), the study was composed of two stages (Fig. 1). Stage A was divided into three periods: (I) 3- to 9-week period during which each patient’s daily r-hGH dose was optimized to achieve a normalization of IGF-I levels between ±1.5 SDS. (II) Weekly MOD-4023 administration for 4 weeks at a dose equivalent to 18.5%, 37%, 55.5% or 123.4% of their own optimized, weekly cumulative molar r-hGH dose from Period I. (III) Up to 50 weeks of maintenance treatment in which patients returned to their optimized dose of daily r-hGH replacement.

The starting dose for Stage B was based on the results from Stage A, Period II. Stage B of the study was divided into two periods: (I) 16-week MOD-4023 treatment in which all patients received the same MOD-4023 dose level (61.7% of their own optimized weekly cumulative molar r-hGH dose from Stage A, Period I), and could have their dose subsequently modified based on IGF-I levels. IGF-I was monitored every 2 weeks, 4 days after dose administration, and patients’ doses were titrated once a month if necessary. (II) 2-week washout and follow-up period, with no r-hGH replacement therapy administered.

The doses for the 4-week treatment period (Stage A, Period II) were initially selected on the basis of molar hGH content of MOD-4023, pre-clinical *in vitro* data including receptor affinity and binding capacity and PK/PD data in relevant animal models (18), as well as the results of the Phase 1 study in healthy adults. As this was the first time MOD-4023 was tested in adults, a wide range of doses were assessed to accommodate the different parameters from the above analysis and to better understand its effectiveness and safety in a clinically relevant population. In the Phase 1 study, the pharmacodynamic responses (IGF-I) of normal adult male volunteers to a single dose of 4, 7 or 21 mg of MOD-4023 were measured and compared to literature data for daily 0.6 mg injections of r-hGH in GHD adults (20). A single 4 mg dose of MOD-4023, compared to daily dosing with 0.6 mg r-hGH, provided approximately twice the total weekly systemic exposure to IGF-I when normalized for the molar content of r-hGH in each regimen. Thus, on a molar basis, a weekly dose of MOD-4023 equivalent to 7 daily injections of r-hGH was initially estimated to be twice as potent as daily r-hGH in inducing IGF-I secretion. In view of that, MOD-4023 doses for Cohorts 1–3 (weekly 18.5%, 37% and 55.5% hGH molar content) were calculated by multiplying the weekly cumulative r-hGH dose (in mg) with the dose level of the specific cohort and with a bioequivalent factor of 0.5 (derived from Phase 1 data). The relatively high MOD-4023 dose level for Cohorts 1A (weekly 123.4% hGH molar content) was also selected as the affinity of MOD-4023 to the GH receptor was shown to be on average 8-fold lower than r-hGH. In addition, this dose was shown to be 3.4-fold higher than that of Cohort 2, thus expanding the range of MOD-4023 doses tested and also providing a higher acute safety margin in case higher doses would be required on an individual basis. Stage A of the study demonstrated that the patients in Cohort 3 (55.5% of the relative molar content of r-hGH) were likely to achieve an appropriate IGF-I response.

After review of the Stage A results, all patients in Stage B received a slightly higher starting dose of approximately 61.7% of the net r-hGH dose to further improve and optimize the weekly PK/PD profile.

### Study protocol

The screening period lasted up to 21 days prior to cohort assignment. In Stage A, Period I (r-hGH treatment optimization), patients with IGF-I levels within ±1.5 SDS (which was set as the target range for this study) continued their established r-hGH dose for 2 weeks until re-evaluation of IGF-I levels in Visit 2. If their IGF-I levels remained within the target range of ±1.5 SDS, the patients underwent cohort assignment 1 week later (Visit 3). Patients whose IGF-I levels were outside ±1.5 SDS but within ±2 SDS had a dose adjustment of their daily r-hGH treatment and were re-tested, for up to 4 re-tests (at Days 28, 42 and 56). Patients with IGF-I values within ±1.5 SDS during optimization were assigned to a study cohort 7 ± 3 days after Visit 3. This visit included collection of blood samples to establish a 36-h r-hGH PK/PD profile (MOD-4023 and IGF-I) as well as fasting glucose/insulin levels.

In Stage A, Period II (active MOD-4023 treatment), the dose administered to each patient was individualized and based on the patient’s assigned cohort and on the cumulative weekly dose of their final daily r-hGH treatment from the optimization period. Patients were treated with MOD-4023 for 4 weeks and received any of the four doses (weekly cohorts) of MOD-4023 subcutaneously by medical staff at the study clinic. If a patient missed an injection, he/she could receive the dose during the following 48 h without skipping that week’s injection and with no potential safety-related concerns based on the MOD-4023 PK/PD weekly profile. Patients allocated to weekly dosing returned to the clinic for the 2nd and 3rd dose administration, where samples for safety assessment, PK/PD, fasting glucose and insulin were taken before and after each MOD-4023 administration. Approximately 12 h before the administration of the last dose (4th dose in weekly cohorts), patients were hospitalized for up to 36 h and blood samples were obtained for PK/PD determination and assessment of glucose metabolism. Weekly cohort patients were assessed for PK/PD and safety at 48 h (Day 2), 72 h (Day 3), 96 h (Day 4), 120 h (Day 5) and 168 h (Day 7) from administration of the last dose. In Stage A, Period III (r-hGH maintenance), the patients returned to their optimized dose of daily r-hGH replacement treatment for 8–50 weeks. They underwent IGF-I levels monitoring and safety assessment on a monthly basis.

Stage B, Period I (16-week MOD-4023 dose titration) included only eligible patients who rolled over from Stage A. Patients started with a weekly MOD-4023 dose level equivalent to 61.7% of the cumulative weekly molar content of daily hGH for 16 weeks. The first MOD-4023 dose was administered by medical staff at the clinic, whereas the rest of the doses were administered by the patients at home. Patients were asked to fill out diary cards on a weekly basis immediately after MOD-4023 injection, describing injected dose, injection site location and pain (if any). For patients who did not achieve normalization of IGF-I, an increment/decrement factor of 33% was used to adjust the MOD-4023 dose. IGF-I, glucose and insulin measurements, as well as safety and immunogenicity assessments, were performed 3–4 days after the 2nd, 4th, 6th, 8th, 10th, 12th, 14th and 16th MOD-4023 dose. In Stage B, Period II (2-week follow-up without r-hGH replacement), samples were taken for measurement of IGF-I anti-MOD-4023 antibody serum levels, and safety assessments were conducted.

### Statistical analysis

Statistical analysis was performed using data from all enrolled patients from all four cohorts who received at least one dose of active treatment. As no data were previously available from adults administered with MOD-4023, a sample size of 12 male patients was chosen, based on considerations of feasibility and precision of estimates for a pilot study (21). IGF-I and IGF-I SDS values are presented by means of summary statistics. The linearity of MOD-4023 PK was assessed using the power model. Comparison of *C*_avg_ between treatments was performed using ANOVA. The geometric mean ratio and confidence intervals were calculated for each cohort using the log-transformed data and the two-sided *t*-test method. All statistical analyses were performed using SAS.

### PK/PD analysis

Serum concentrations of MOD-4023 were measured using a validated quantitative sandwich enzyme immunoassay (Intertek Analytical Laboratory, El Dorado Hills, CA, USA). Serum concentrations of IGF-I were centrally measured and analyzed at the Endocrine Laboratory, Medizinische Klinik und Poliklinik IV, Klinikum der Universität (Munich, Germany) using an automated chemiluminescence assay (IDS iSYS, Immunodiagnostic Systems, Boldon, UK). The IGF-I SDS values were calculated on the basis of assay-specific IGF-I reference intervals matched for age and gender (22). PK/PD analysis for Cohorts 1A, 2 and 3 were performed by István Kovács (Accelsiors, Budapest, Hungary). Additional analyses were performed by William G Kramer (Kramer Consulting, North Potomac, MD, USA) using WinNonLin Professional, v5.3.

### Safety evaluation

Safety assessments consisted of monitoring and recording all AEs including serious adverse events (SAEs), vital signs, physical condition and body weight. Safety evaluation also included fundoscopy, glucose metabolism (HbA1c, fasting insulin and glucose levels), thyroid hormones (free T4 and TSH), cortisol levels, laboratory parameters (serum chemistry, liver enzymes, hematology, lipoproteins and urinalysis), local tolerability and immunogenicity. Injection site pain was assessed by the patients and recorded in their diary after each injection using a validated Visual Analog Scale (23).

### Antibody assessments

Patients were tested at screening for antibodies against r-hGH and again at the end of Stage A using a radio-precipitation assay. The presence of serum antibodies against MOD-4023 was evaluated in Stage A, Period II before the 4th dose, and at the beginning and end of the 4-week treatment period with MOD-4023. One week prior to patients starting Stage B, Period I (MOD-4032 treatment), serum was tested for anti-hGH autoantibodies. Serum samples were screened for the presence of anti-MOD-4023 using a validated qualitative electrochemiluminescence (ECL) assay. Briefly, samples were co-incubated with biotin-MOD-4023 and Sulfo-Tag-MOD-4023 in polypropylene tubes. The samples were subsequently added to a streptavidin plate (Meso Scale Diagnostics, Rockville, MD, USA) and incubated. After incubation, unbound material was washed away and anti-MOD-4023 antibodies were detected using Sector Imager 2400 (Meso Scale Diagnostics). Positive samples in the screening assay were titrated and subsequently tested in a second, confirmatory assay for MOD-4023 specificity, as well for binding specificity to the hGH or CTP domains of MOD-4023 using immuno-competition or bridging ELISA respectively. Finally, antibodies showing specificity for MOD-4023 or hGH were evaluated in a qualitative cell-based assay for neutralizing activity (inhibition of MOD-4023- or hGH-induced cell proliferation). Briefly, patient samples were incubated with 25 ng/mL MOD-4023 at room temperature. Human BaFB2B cells were added and incubated for 18 ± 2 h at 37°C + 5% CO_2_. 30 μL of CellTiter96 AQueous One solution (Promega) were subsequently added. Measured A490 is proportional to the living cell count and inversely proportional to the level of neutralizing antibodies.

## Results

### Patient disposition

Fifty-four Caucasian patients (8 females and 46 males) from 15 centers in six countries were enrolled and randomized into Cohorts 1–4. Patient characteristics are summarized in Table 1. Three patients were enrolled into the study but discontinued prior to receiving any treatment. Thirty-one patients (57%) had adult-onset GHD, whereas 23 (43%) had GHD of childhood onset. A total of 43 patients continued to Stage B of the study. There were 12 premature study discontinuations throughout the study: four patients were unwilling to proceed to Stage B; one patient was unable to continue the study; one patient did not achieve the required IGF-I levels after treatment with MOD-4023; there were no additional findings that could potentially explain this observation (e.g. no anti-MOD-4023 antibodies). One patient was discontinued due to an unrelated AE (myocardial ischemia), and five patients discontinued for other reasons. A total of 42 patients completed Stage B.

**Table 1:** Summary of baseline characteristics.

	Stage A				Stage B
	Cohort 1 weekly 18.5% molar content* (n = 3)**	Cohort 2 weekly 37% molar content* (n = 13)	Cohort 3 weekly 55.5% molar content* (n = 13)	Cohort 1A weekly 123.4% molar content* (n = 13)	Weekly 61.7% (n = 43)
Age (years)					
Mean (±s.d.)	44.3 (±17.7)	40.9 (±13.0)	41.5 (±10.8)	40.5 (±11.1)	42.7 (±11.6)
Median	53.0	39.0	41.0	38.0	42.0
Range	24–56	25–60	25–58	23–59	23–60
Gender, n (%)					
Male	3 (100%)	11 (85%)	11 (85%)	11 (85%)	35 (81%)
Female	0 (0%)	2 (15%)	2 (15%)	2 (15%)	8 (19%)
Body weight (kg)					
Mean (±s.d.)	97.3 (±13.6)	89.6 (±17.2)	81.5 (±17.4)	78.4 (±15.7)	83.7 (±16.1)
Median	102	90.0	88.0	75.5	81.0
Range	82–108	63–121	54–101	53–109	54–121
BMI (kg/m2)					
Mean (±s.d.)	30.1 (±4.0)	29.3 (±4.1)	28.3 (±4.6)	26.6 (±4.1)	28.2 (±4.0)
Median	30.1	27.7	29.2	26.4	28.8
Range	26.2–34.1	24.2–34.9	20.6–34.7	19.5–34.2	20.6–34.2

* Weekly molar equivalent of daily r-hGH. **Discontinued cohort.

### Pharmacokinetics

**Figure 2:**
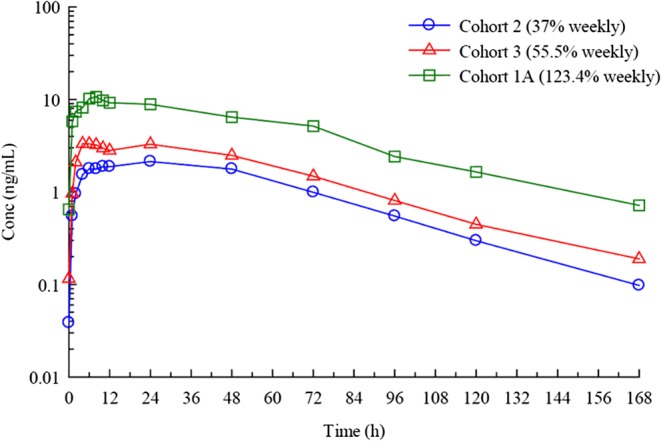
Pharmacokinetic analysis of MOD-4023 administered in GHD adults. Mean plasma concentrations of MOD-4023 after SC administration weekly to GHD adults. MOD-4023 doses are based on protein content.

The once-weekly dose of MOD-4023 during Stage A ranged from 0.26 to 10.71 mg. In all patients, the trough levels of MOD-4023 in all instances were clearly below 5% of *C*_max_; MOD-4023 levels ranged from below the detection limit of <0.15 ng/mL to 2.65 ± 2.77 ng/mL (Table 2). Cohort 2 had a higher average daily r-hGH dose than Cohort 3, leading to similar MOD-4023 doses. Patients in Cohort 1A received a higher mean r-hGH dose during the optimization period than Cohorts 2 or 3 and received a MOD-4023 dose equivalent to 123.4% of the weekly cumulative dose of r-hGH. As a result, nine of the 13 patients in Cohort 1A had an absolute MOD-4023 dose that was higher than that for any patient in Cohorts 2 and 3. There was a dose-related increase in the mean plasma concentrations and mean values of *C*_max_ and AUC_(0−_*_τ_*_)_ from Cohort 2 to Cohort 3 to Cohort 1A, representing molar equivalents of 37%, 55.5% and 123.4% of daily r-hGH (Fig. 2). Log–log plots (power model) of the individual patient values for *C*_max_ and AUC_(0−_*_τ_*_)_ indicated linear pharmacokinetics over a ~40-fold dose range (not shown). The mean values for clearance, volume of distribution and *t*_1/2_ were comparable for all three cohorts, with no apparent trends (Table 2).

**Table 2:** Summary of pharmacokinetic parameters for MOD-4023 (±s.d.).

Parameter*	Cohort 2 weekly 37% molar content	Cohort 3 weekly 55.5% molar content	Cohort 1A weekly 123.4% molar content
Dose (mg)	1.23 ± 0.57	1.29 ± 0.67	5.62 ± 3.11
C_max_ (ng/mL)	2.65 ± 2.77	4.06 ± 4.27	14.4 ± 14.71
T_max_ (h)	12	10	8
C_min_ (ng/mL)	0.038 ± 0.13	0.106 ± 0.24	0.638 ± 1.39
AUC_(0−τ)_ (h × ng/mL)	160 ± 186	245 ± 303	717 ± 813
C_avg_ (ng/mL)	0.954 ± 1.11	1.46 ± 1.80	4.27 ± 4.84
Percent fluctuation	289 ± 58.1	329 ± 82.2	343 ± 210
λ_z_ (1/h)	0.0222 ± 0.0055	0.0217 ± 0.0054	0.0200 ± 0.0051
t_1/2_ (h)	32.7 ± 6.42	34.0 ± 8.92	36.9 ± 9.92
CL/F (mL/h)	13 570 ± 10 181	11 459 ± 7219	12 505 ± 6061
V_z_/F (mL)	674 904 ± 625 819	573 597 ± 463 706	671 468 ± 407 033

* *λ*_z_, terminal elimination rate; AUC, area under curve; CL/F, apparent total clearance; *C*_avg_, average concentration; *C*_max_, maximal concentration; *C*_min_, minimal concentration; *t*_1/2_, half-life; *T*_max_, time of maximal concentration; *V*_z_/F, apparent volume of distribution.

Comparisons between exposure to hGH after administration of r-hGH (Period I) and MOD-4023 (Period II) are based on net hGH content (72.6% of the MOD-4023 molecule) and *C*_avg_, which is normalized for the dosing interval. *C*_avg_ achieved after administration of MOD-4023 was comparable to that of r-hGH for both Cohorts 2 and 3 (37% and 55.5% of molar r-hGH content respectively). The arithmetic mean *C*_avg_ after administration of MOD-4023 to Cohort 1A (weekly molar equivalent of daily r-hGH) was ~2-fold higher than that after administration of r-hGH (1.27 ± 1.41 ng/mL). The geometric means were 1.91 and 0.78 ng/mL with a geometric mean ratio of 243.24% (data not shown). This indicates that administration of the molar equivalent of 123.4% of daily r-hGH dose results in an approximate 2.4-fold increase in hGH exposure.

### Pharmacodynamics

**Figure 3:**
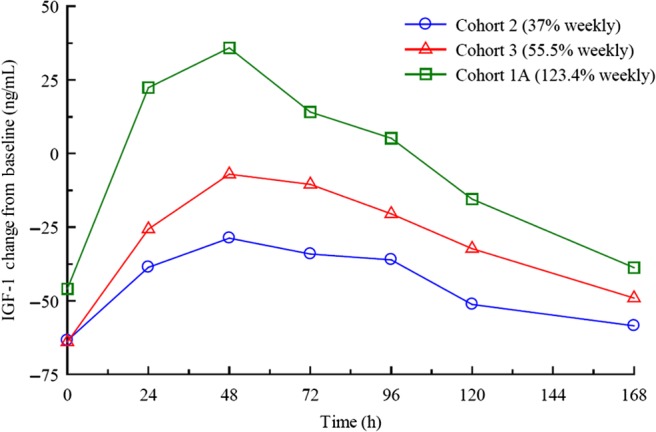
Pharmacodynamic analysis of MOD-4023 in GHD adults. Mean change from baseline IGF-I plasma concentrations after weekly MOD-4023 administration. The mean increase in IGF-I was calculated for each time point by subtracting the IGF-I concentration at each time point from the baseline IGF-I value from the end of the r-hGH run-in period.

IGF-I is a surrogate serum marker for hGH treatment efficacy in GHD adults while also serving as a safety parameter in monitoring GH replacement therapy to avoid overdosing. Baseline IGF-I plasma concentrations for cohorts administered with weekly MOD-4023 doses equivalent to 37% (Cohort 2), 55.5% (Cohort 3) and 123.4% (Cohort 1A) of the daily r-hGH dose were 160 ± 47.7, 174 ± 52.6 and 135 ± 37.0 µg/L respectively. Once-a-week administration of MOD-4023 exhibited a dose-dependent response when calculating mean change from baseline IGF-I concentrations (Fig. 3). IGF-I *C*_avg_ values after administration of r-hGH were comparable among the three weekly dosing cohorts. After administration of MOD-4023 at a dose equivalent to 37% of the cumulative daily r-hGH dose (Cohort 2), there was a decrease in IGF-I *C*_avg_ with a geometric mean ratio of 68.06%. Administration of MOD-4023 at 55.5% (Cohort 3) and 123.4% (Cohort 1A) of the cumulative daily r-hGH dose resulted in IGF-I *C*_avg_ values that were comparable to those obtained after daily administration of r-hGH. The geometric mean ratios (MOD-4023 to r-hGH) were virtually identical (Table 3).

**Table 3:** Statistical comparison of average IGF-I concentrations after subcutaneous administration of r-hGH and MOD-4023.

	Geometric means		Geometric mean ratio (%)	
Cohort	MOD-4023	r-hGH	Estimate	90% confidence interval
2 (37% weekly)	111.99	164.54	68.06	51.43 → 90.06
3 (55.5% weekly)	138.94	172.87	80.37	68.06 → 94.91
1A (123.4% weekly)	120.02	151.63	79.15	61.20 → 102.38

**Figure 4:**
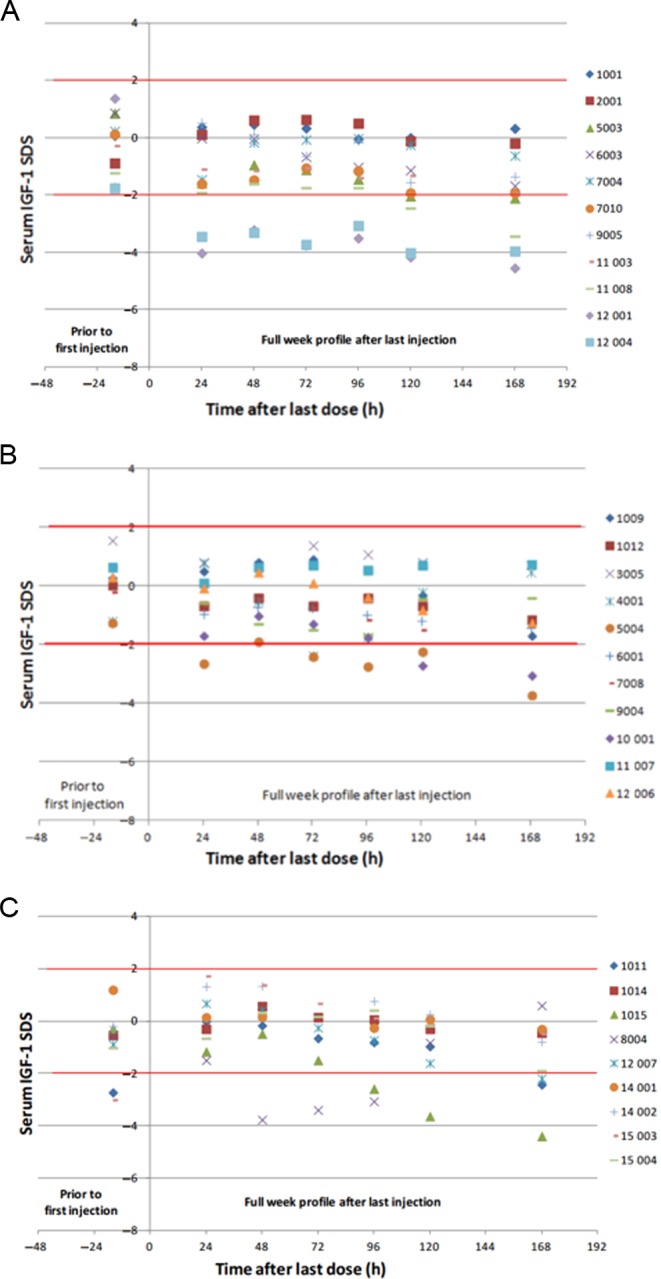
IGF-I SDS after 4th weekly dose of MOD-4023 (Stage A). Serum IGF-I SDS values are shown for male patients from Cohort 2 (37% of r-hGH content; panel A), Cohort 3 (55.5% of r-hGH content; panel B) and Cohort 1A (123.4% of r-hGH content; panel C).

For Cohort 2 (weekly molar equivalent of 37% of daily r-hGH dose), the IGF-I values of 5 out of 9 subjects were >−2 SDS 168 h after injection (Fig. 4A). *E*_avg_ was −1.483 ± 1.384, within the normal range of ±2 SDS and also within the target range of ±1.5 SDS, suggesting that IGF-I levels were within that range for most of the weekly treatment period. For Cohort 3 (weekly equivalent of 55.5% of daily r-hGH), the majority of IGF-I values fell within ±2 SDS (*E*_avg_ = −0.567 ± 1.087) and most of those were within the target of ±1.5 SDS during the weekly treatment period (Fig. 4B). Cohort 1A (equivalent of 123.4% of daily r-hGH) had an average IGF-I SDS baseline lower than Cohorts 2 and 3, and a *E*_avg_ value of −1.348 ± 2.180 (Fig. 4C).

**Figure 5:**
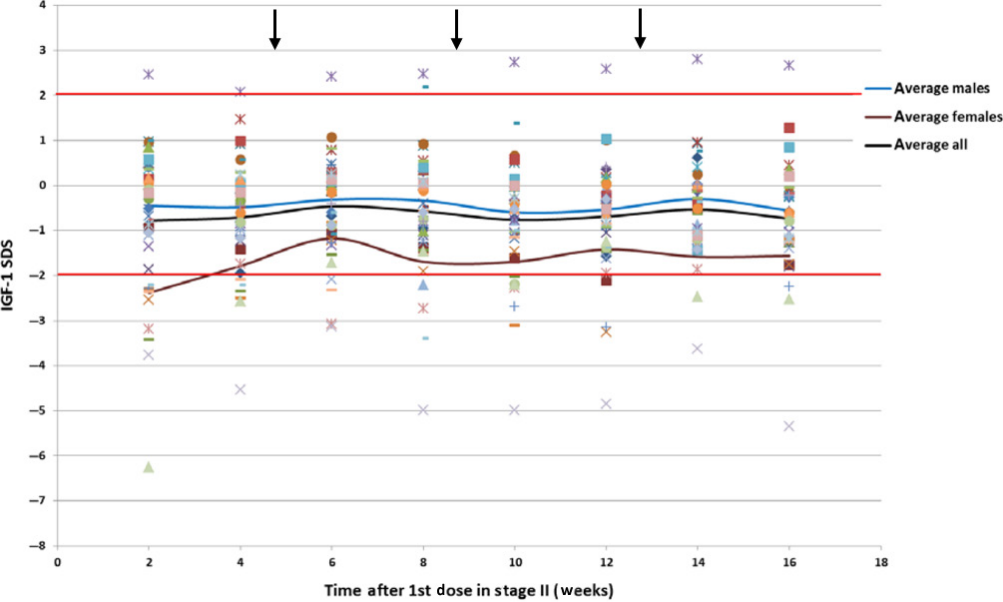
IGF-I SDS levels during Stage B. Patients were administered weekly doses of MOD-4023 equivalent to 61.7% of the relative molar content of daily r-hGH. IGF-I measurements were performed 4 days after MOD-4023 administration. The arrows indicate the times at which dose titration was performed if needed.

After Stage A, Period III (in which patients returned to r-hGH replacement treatment), patients that participated in Stage B of the study received weekly doses of MOD-4023 equivalent to 61.7% of the daily r-hGH dose for 16 weeks. IGF-I assessment was performed 4 days after administration of MOD-4023. As shown in Fig. 5, most of the patients maintained stable IGF-I levels within ±2.0 SDS throughout Stage B. When required, MOD-4023 dose levels were titrated up or down by 33% of the starting dose based on patients’ IGF-I levels. Fourteen patients required increases in dose. Six patients had a single increase in dose, five patients received two increases in dose and three patients received three dose increases. 12 of the 17 patients with dose modifications achieved normal IGF-I levels (SDS within ±2.0) by the end of MOD-4023 dosing. Three patients required reductions in MOD-4023 dose. Two of these patients achieved IGF-I normalization by the end of the MOD-4023 treatment period. No accumulation of IGF-I was observed during the 16-week treatment period.

The study determined that weekly MOD-4023 doses maintained IGF-I levels within the normal range in the majority of patients. In Stage A, the mean time intervals with IGF-I levels that lay within ±1.5 SDS after MOD-4023 administration were not significantly different across the three weekly dose levels, as all mean time intervals were within one standard deviation of one another. IGF-I levels in Cohort 2 (weekly molar equivalent of 37% of daily r-hGH) were within ±1.5 SDS for a mean of 93 h and within ±2 SDS for a mean of 121 h. The mean IGF-I levels of patients in Cohort 3 (weekly equivalent of 55.5% of daily r-hGH) were within ±1.5 SDS for a mean of 139 h and within ±2 SDS for a mean of 146 h (87% of the week). IGF-I levels of patients in Cohort 1A (weekly equivalent of 123.4% of daily r-hGH) were within ±1.5 SDS for a mean of 108 h and within ±2 SDS for an average of 120 h, although this cohort started with relatively lower IGF-I baseline levels.

### Safety

#### Adverse events

Overall, 29 of the 54 patients (53.7%) experienced at least one AE during daily r-hGH treatment (Stage A, Period I or III). Five patients (9.3%) experienced a total of five serious adverse events (SAEs; AEs that result in hospitalization, disability or are life threatening) during daily r-hGH treatment. During Stage A, Period II (MOD-4023 treatment), 22 patients (40.7%) experienced at least one AE; there were no severe AEs (i.e., AEs that cause severe discomfort and limit the subject’s normal activities) and no SAEs. There were no AEs leading to MOD-4023 treatment withdrawal during Stage A of the study. Of the 40 AEs reported during Period II, 16 events were assessed as possibly or probably related to MOD-4023. Two events of headache were assessed as probably related to MOD-4023 treatment (Table 4). During Stage B (MOD-4023 treatment), a total of 18 of the 43 patients (41.9%) experienced at least one AE. Overall for Stage B, AEs were reported for 20 patients (46.5%). Three patients (7.0%) experienced a total of four unrelated SAEs (myocardial ischemia, infectious diarrhea, gastroenteritis and amnesia). Six patients (14.0%) experienced severe events and one patient (1.9%) discontinued due to an unrelated AE. Four patients (9.3%) experienced at least one AE during the 2-week washout period of Stage B, Period II. Of the total of 47 AEs reported during this period, two events of headache were assessed as possibly related to MOD-4023. Three AEs (in two patients) were considered to be related to MOD-4023 (increased blood glucose and free thyroxine and decreased hemoglobin (Table 4)). No SAEs that occurred during the study were considered to be related to MOD-4023 administration (infectious diarrhea, gastroenteritis, amnesia, aspiration and myocardial ischemia).

**Table 4:** Summary of adverse events possibly or probably related to MOD-4023 treatment.

	Stage A			Stage B
	Cohort 2 37% weekly	Cohort 3 55.5% weekly	Cohort 1A 123.4% weekly	61.7% weekly
No. of patients	13	13	13	43
No. of patients with any AEs	5	3	6	20
No. of AEs	1	3	8	5
Nausea	0	0	1	0
Fatigue	0	0	1	0
Pain in extremity	0	0	1	0
Headache	0	0	3	2
Dizziness	0	0	1	0
Insomnia	0	0	1	0
Face edema	0	1	0	0
Peripheral edema	0	1	0	0
Hypertension	0	1	0	0
Increased blood glucose	0	0	0	1
Increased glycosylated hemoglobin	0	0	0	0
Decreased hemoglobin	0	0	0	1
Decreased WBC count	1	0	0	0
Increased free thyroxine	0	0	0	1

#### Injection site reactions

Injection site reactions were noted in three patients in Stage A (one case of short-lasting pain and two cases of mild erythema), all of which resolved over several days without intervention. In Stage B, three patients developed mild (2)-to-moderate (1) lipoatrophy. It was of transient nature and resolved with adequate rotation of injection sites within 35–60 days after the last MOD-4023 dose was administered to the area. All three patients continued MOD-4023 treatment and successfully completed the study.

#### Antibody assessments

None of the patients tested positive for pre-existing anti-hGH antibodies. Anti-MOD-4023 antibodies were found in one patient at the end of Stage A. Five patients were found positive for binding anti-MOD-4023 and anti-hGH antibodies at the end of Stage B at very low titers. None of these patients was confirmed positive for anti-CTP antibodies, and no anti-MOD-4023 or anti-hGH neutralizing activity was detected. In Stage B, IGF-I levels clearly stayed within the normal range of above −2 SDS in these six patients.

## Discussion

MOD-4023 is a novel human GH modified with CTP technology to extend the duration of its activity. Enhancing the protein’s biopotency allows administration on a weekly basis. This is in contrast to the currently available daily r-hGH formulations; weekly GH may reduce discomfort and increase medication adherence and quality of life. Unlike other long-acting GH products in development, the CTP moiety of MOD-4023 is derived from a natural peptide, the C-terminal peptide of hCG, and as such is likely to be less immunogenic than other moieties. The present Phase 2 clinical trial assessed the safety, tolerability and PK/PD profile of three MOD-4023 doses in GHD adults. The study consisted of two stages: Stage A evaluated the effectiveness and PK/PD profile of four dosing regimens of MOD-4023 in GH-deficient adults after their IGF-I levels were optimized on daily r-hGH therapy to guide dose selection. Stage B was a 16-week treatment period intended to confirm the selected MOD-4023 therapeutic range to be used for the Phase 3 study.

Stage A PK data for MOD-4023 across the weekly dosing cohorts investigated during this study confirmed a dose-response, with linear pharmacokinetics over a 40-fold dose range as determined by analysis of *C*_max_ and AUC_(0−_*_τ_*_)_ values. No apparent accumulation of MOD-4023 was observed throughout the study duration. A similar dose–response correlation was observed in analysis of the PD data. It is possible that the 123.4% dose level (Cohort 1A) could have had a longer duration of efficacy than the 55.5% dose (Cohort 3), but interpretation of the efficacy data for this cohort was confounded by lower baseline IGF-I levels than the other dosing cohorts.

IGF-I is a surrogate marker in GH treatment used for hGH dose monitoring and for the predictability of treatment efficacy (24). Based on established guidelines for the use of growth hormone (1) and other published work, achievement of optimal favorable effects on body composition requires maintaining IGF-I SDS within the normal range (25, 26). In a prior study of a long-acting GH (27), administration of a weekly compound (LB03002) for six months led to an IGF-I SDS mean value at the study endpoint (−0.43; s.d. 1.98) that was similar to the mean obtained in Stage B of the present study. Subjects in that study experienced significant reductions in mean fat mass as compared to the placebo group.

One issue when dosing long-acting GH is to determine the optimal timing to draw blood for measurement of IGF-I; this is important because dose adjustments are made based on the level of this hormone. IGF-I *C*_avg_ determined during Stage A, Period II was shown to predominantly correlate to IGF-I mean values 96 h (4 days) after administration of MOD-4023. Therefore, this time point was selected for monitoring of IGF-I during Stage B of the study.

Evaluation of IGF-I levels in Stage B was performed four days after dosing to coincide with the time MOD-4023 *C*_avg_ correlated to the mean IGF-I values, as determined in the PD analysis of Stage A, Period II. The majority of subjects (88%) achieved normalization of IGF-I (i.e., IGF-I SDS within ±2.0) by the time of the final Stage B post-dose evaluation. Two exceptions to this observation were 2 patients whose IGF-I SDS levels remained above 2 and around −5 throughout Stage B. Possible explanations that were explored for these IGF-I SDS levels were acromegaly in the former and GH resistance in the latter, but this was not confirmed. The majority of patients (62%) did not require dose modifications, and most of the patients who did need a change in dose achieved normalization by the end of Stage B. Because normalization was achieved in the majority of the patients using a dose that was lower than the total amount administered over 7 days of their optimized daily hGH, transitioning patients from daily therapy to weekly MOD-4023 administration may reduce the extended overall exposure to hGH. It is interesting to speculate whether this might have positive safety implications, especially in light of the chronic nature of this indication. Overall, based on Stage A and B of this study, the proposed MOD-4023 dose range is approximately 1.23–5.62 mg (equivalent to 37–123.4% molar content of MOD-4023). This study provided data to guide the design of a pivotal Phase 3 study.

MOD-4023 exhibited highly favorable safety profiles for all dosing cohorts, with no unexpected AEs considered to be related to MOD-4023. Relatively few AEs were attributed to MOD-4023 overall, and those potentially related AEs that were reported were similar to those expected for r-hGH treatment (28, 29, 30, 31). There was only a single case of injection pain; of note, the needle size (31 g) used for the administration of MOD-4023 was similar to that used for daily r-hGH applications, and smaller than that in other sustained-release GH formulations such as LB03002 or Nutropin Depot. The 3 lipoatrophy cases reported in Stage B may have been due to insufficient rotation of injection sites in the abdomen, as they resolved once different sites were used. Data about lipoatrophy are being carefully monitored in ongoing trials and will be further evaluated in future studies. No accumulation of IGF-I was observed over the studied period, with IGF-I exceeding +2 SDS in only one patient during Stage B. This appeared to be GH independent as IGF-I levels were not reduced after reduction in dose. After five months of MOD-4023 administration, anti-MOD-4023 antibodies were observed only in five patients. Antibody levels were very low in these patients and were not associated with neutralizing activity. The absence of neutralizing activity in patients positive for binding antibodies was also corroborated by the IGF-I levels measured throughout the study. Antibody results of this study are consistent with observations made in studies of other r-hGH products (32, 33, 34, 35, 36).

In conclusion, based on the results of the present Phase 2 study, MOD-4023 may obviate the need for daily injections for the treatment of GHD. The results demonstrate that MOD-4023 can be injected once per week in a dose that maintains stable IGF-I level compared to daily GH and holds promise for achieving clinical efficacy while maintaining a favorable safety profile. Less-frequent injections may improve GH replacement adherence and potentially overall outcome. The promising results achieved with MOD-4023 during this study led to a pivotal Phase 3 clinical trial, which is currently ongoing.

## Declaration of interest

C J S, M B and B M K B are members of a medical advisory board for OPKO Biologics. E F, R K, L A, O H and G H are employees of OPKO Biologics.

## Funding

This research did not receive any specific grant from any funding agency in the public, commercial or not-for-profit sector.
